# Supplementary material for: serosim: An R package for simulating serological data arising from vaccination, epidemiological and antibody kinetics processes
Source: PLoS Comput Biol. 2023 Aug 14;19(8):e1011384. doi: 10.1371/journal.pcbi.1011384 (PMC10449138; doi:10.1371/journal.pcbi.1011384)
Supplement: S1 Text — (DOCX) [file pcbi.1011384.s001.docx]

**Supplementary Text**

**S1 Text. Helpful functions used to generate inputs for runserosim**

**S1.1 Text.** **generate_pop_demography:** This function helps users build their demography tibble with birth times, removal times, and specific demographic elements of interest. This function calls both the **simulate_birth_times** and **simulate_removal_times** functions which simulate random birth times and removal times for each individual. See the **generate_pop_demography** help file for more information on how to specify the limits and probabilities of birth and removal times. With the aux in **generate_pop_demography**, users can specify other demographic elements of interest by passing through a list of the column name, options and distributions for each option type. The returned demography tibble can be modified post-hoc to use user-specified distributions and values.

**S1.2 Text.** **reformat_biomarker_map**: This function will reformat the biomarker_map or model_pars objects so that exposure_ID and biomarker_ID are either both numeric (if passed as characters) or characters (if passed as numeric) (See S1 Fig). **runserosim** requires that both exposure_ID and biomarker_ID are numeric entries.

**S1.3 Text. plot_biomarker_mediated_protection:** If the user has specified an immunity model with biomarker mediated-protection, they can use this function to produce a plot of the probability of infection given an individual's biomarker quantity at exposure conditional on biomarker_prot_midpoint and biomarker_prot_width which are specified within model_pars. Ready-to-use immunity models which incorporate biomarker mediated protection are immunity_model_ifxn_biomarker_prot and immunity_model_vacc_ifxn_biomarker_prot (S5 Table). Users can use this function to determine the best values for these two inputs within model_pars.

**S1.4 Text. plot_biomarker_dependent_boosting:** If the user has specified a function to draw parameters with biomarker quantity dependent boosting effects, they can use this function to produce a plot displaying the proportion of full boost received at each starting biomarker quantity given the biomarker quantity ceiling threshold and the biomarker quantity ceiling gradient which are specified within model_pars. Ready-to-use draw_parameters functions which incorporate biomarker dependent boosting are draw_parameters_fixed_fx_biomarker_dep and draw_parameters_random_fx_biomarker_dep (S7 Table). Users can use this function to determine the best values for these two inputs within model_pars.
