# Supplementary figures and images for: serosim: An R package for simulating serological data arising from vaccination, epidemiological and antibody kinetics processes
Source: PLoS Comput Biol. 2023 Aug 14;19(8):e1011384. doi: 10.1371/journal.pcbi.1011384 (PMC10449138; doi:10.1371/journal.pcbi.1011384)

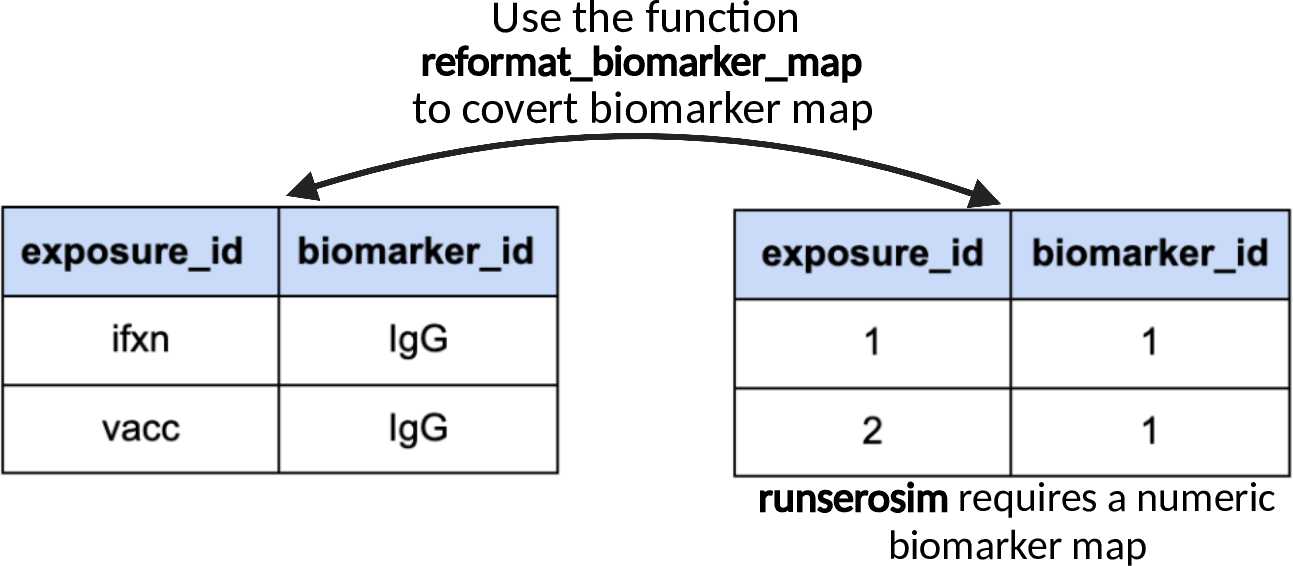

Supplement: S1 Fig — The runserosim function requires a numeric biomarker map input as seen on the right. In this example, we have two exposure events (exposure_id = ifxn and vacc) and we are interested in tracking one biomarker (biomarker_id = IgG) produced by both exposure events. Created with BioRender.com. (TIF) [file pcbi.1011384.s002.tif]

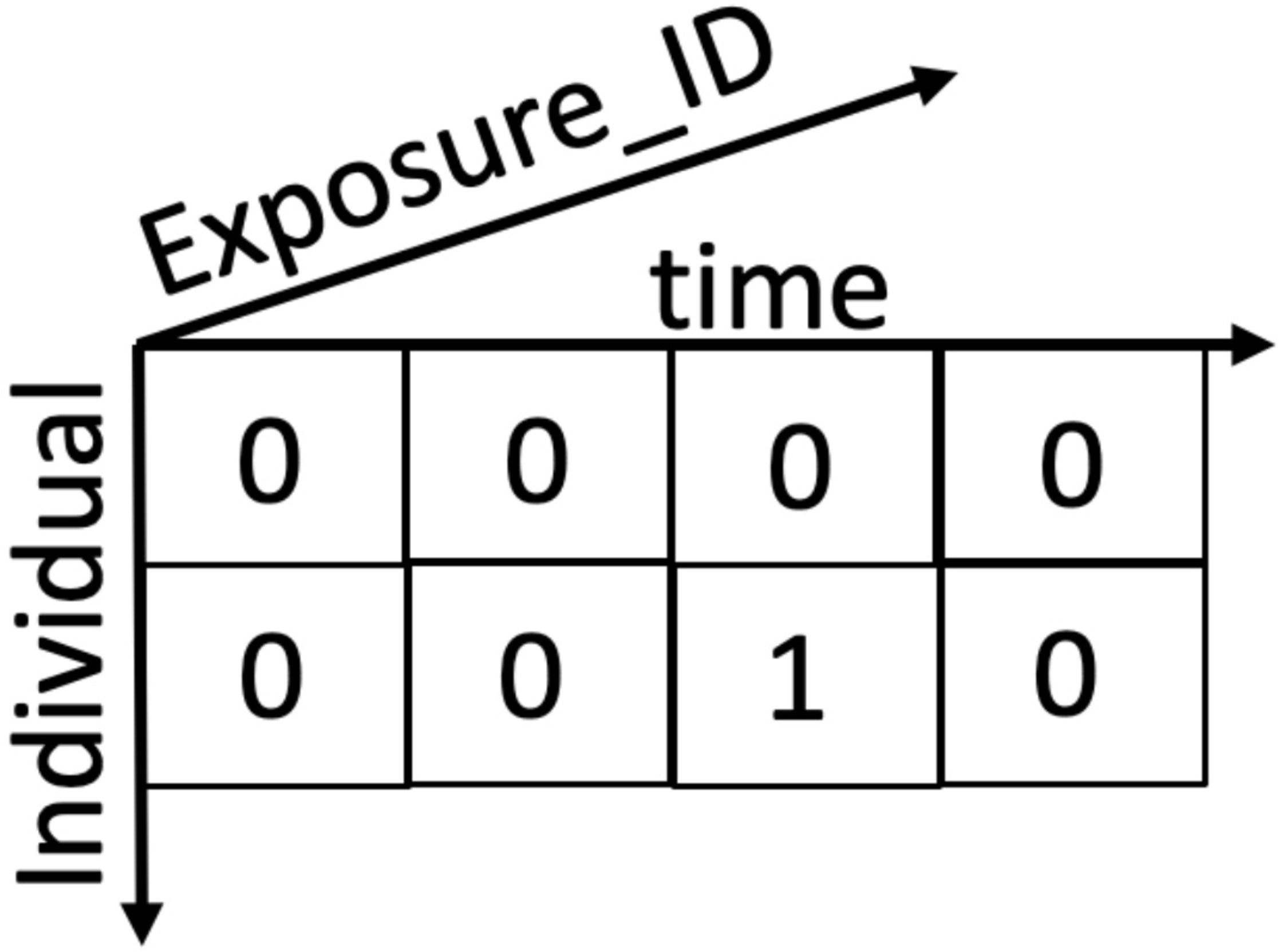

Supplement: S2 Fig — The immune_histories argument is a 3-dimensional array indicating the individual (dimension 1) at each time (dimension 2) for each exposure event (dimension 3). Here, individual 1 never had a successful exposure while individual 2 was exposed at time 3. (TIF) [file pcbi.1011384.s003.tif]

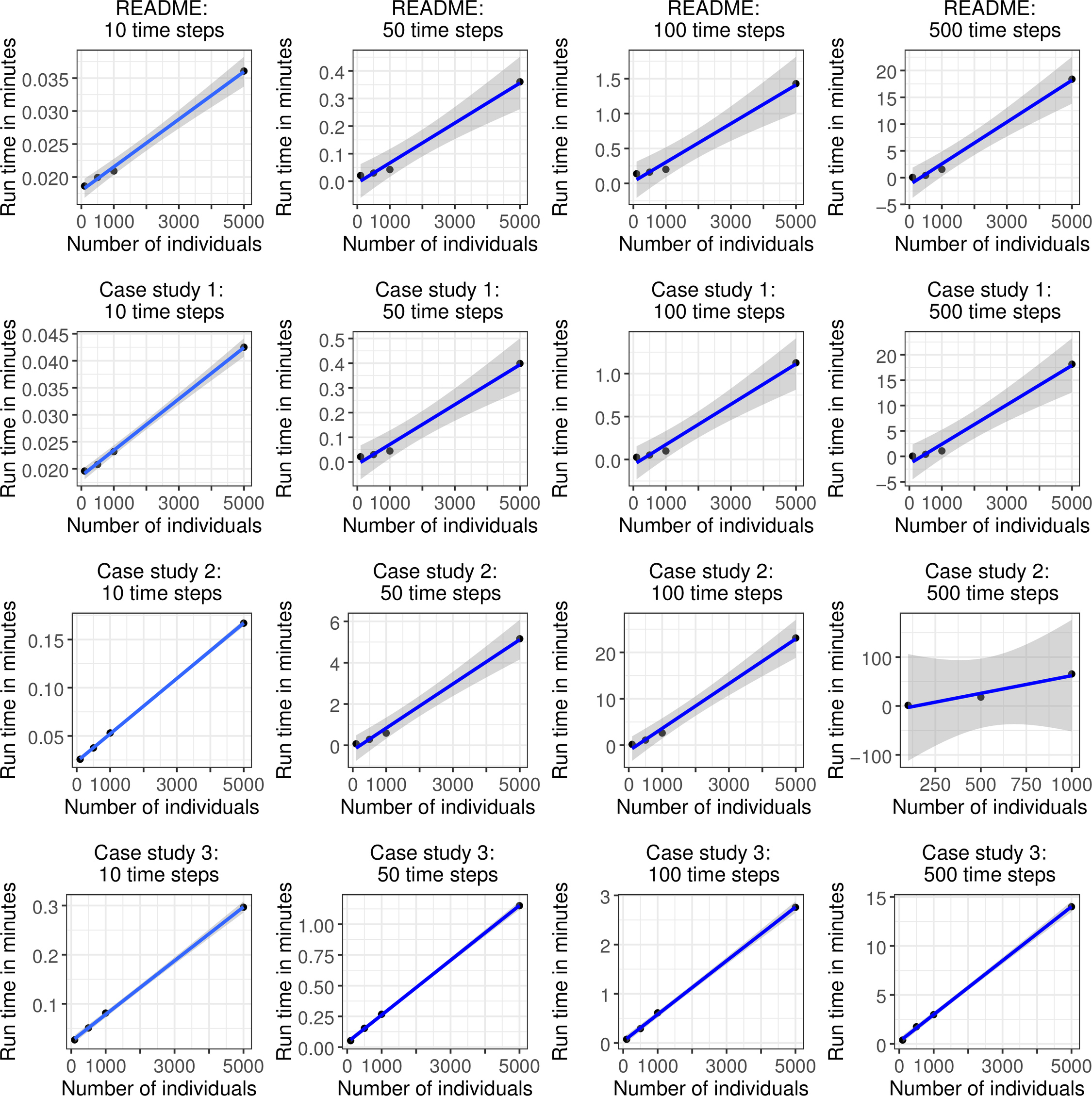

Supplement: S3 Fig — We ran the runserosim function 100 times and report the mean run times under various simulation settings (number of individuals and time steps). Both parallelization and pre-computation within runserosim were turned on and 8 cores were specified. Each case study varies in complexity (S9 Table). The blue line represents a simple linear regression (run time ~ number of individuals) and the gray shaded region is the 95% confidence interval. Simulation run time scaled linearly with increases in the number of individuals. (TIF) [file pcbi.1011384.s004.tif]

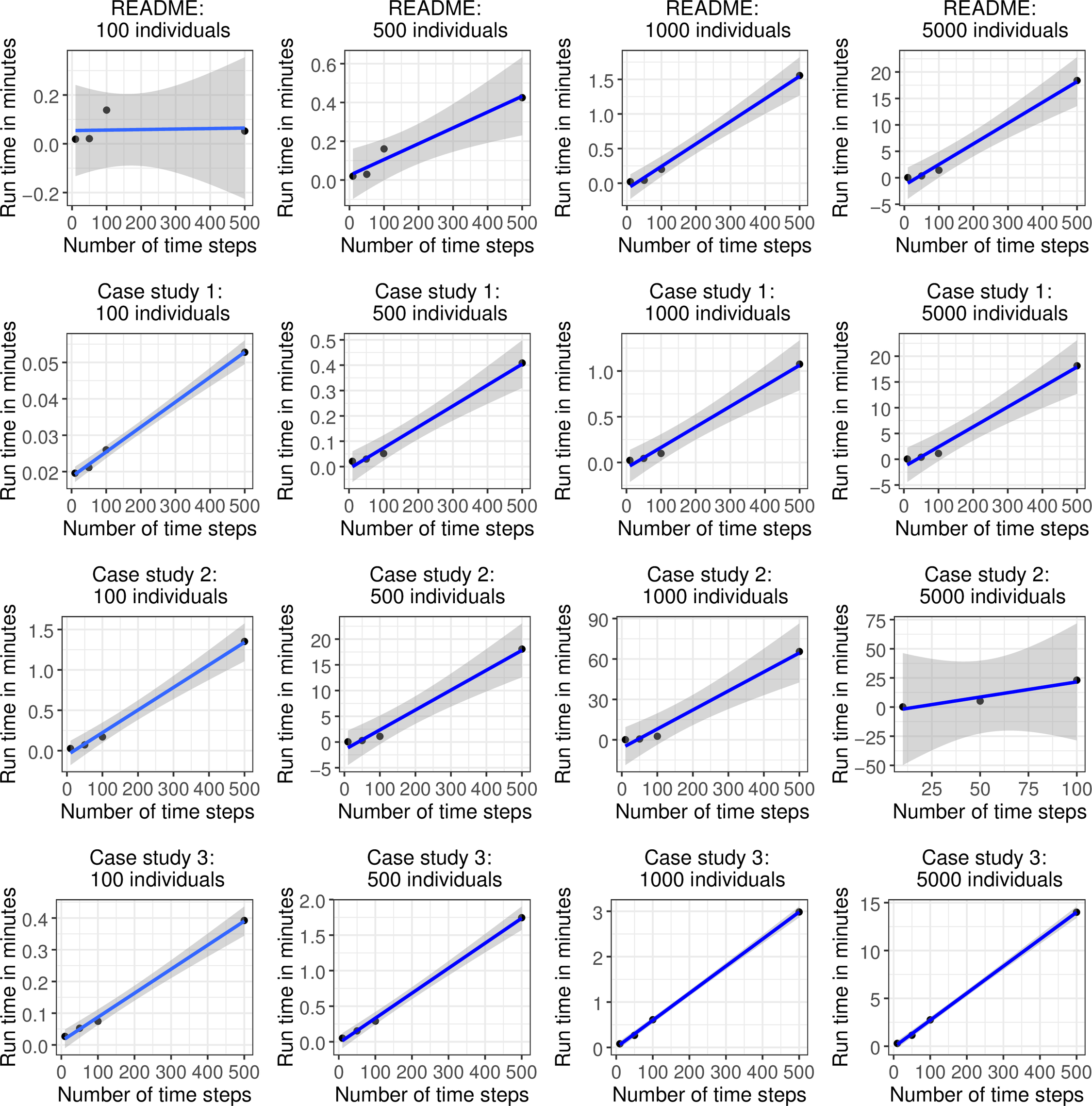

Supplement: S4 Fig — We ran the runserosim function 100 times and report the mean run times under various simulation settings (number of individuals and time steps). Both parallelization and pre-computation within runserosim were turned on and 8 cores were specified. Each case study varies in complexity (Table S9). The blue line represents a simple linear regression (run time ~ number of time steps) and the gray shaded region is the 95% confidence interval. Simulation run time scaled linearly with increases in the number of time steps. (TIF) [file pcbi.1011384.s005.tif]
